# Application of Synthetic Layered Sodium Silicate Magadiite Nanosheets for Environmental Remediation of Methylene Blue Dye in Water

**DOI:** 10.3390/ma10070760

**Published:** 2017-07-06

**Authors:** Mohamed Mokhtar

**Affiliations:** 1 Chemistry Department, Faculty of Science, King Abdulaziz University, P.O. Box 80203, Jeddah 21589, Saudi Arabia; mmokhtar2000@yahoo.com or mmoustafa@kau.edu.sa; Tel.: +966-26-194-983; Fax: +966-26-952-292; 2 Physical Chemistry Department, National Research Centre, El Buhouth St., Dokki, Cairo 12622, Egypt

**Keywords:** adsorption, magadiite, cationic clay, methylene blue, remediation

## Abstract

The removal of methylene blue (MB) dye from water was investigated using synthetic nano-clay magadiite (SNCM). SNCM was synthesized by a hydrothermal treatment under autogenous pressure. A rosette-shaped single mesoporous magadiite phase with 16.63 nm average crystallite size and 33 m^2^∙g^−1^ Braunauer-Emmet-Teller (BET)-surface area was recorded. The adsorption results indicated the pronounced affinity of the SNCM to the MB dye molecules, which reached an adsorption uptake of 20.0 mg MB dye/g of SNCM. The elimination of MB dye by the SNCM was kinetically and thermodynamically considered; a pseudo-second-order kinetic model was attained, and its spontaneous, chemical, and endothermic nature was verified. SNCM was shown to be robust without a detectable reduction in the adsorption capacity after up to four times re-use.

## 1. Introduction

Water pollution is one of the most serious problems that threatens the life of humankind and other living organisms on Earth. This is mainly due to the extensive industrialization of all aspects of our lives, and the spreading of all classes of pollutants in the ecosystem. Organic pollutants are one of the most dangerous classes of pollutant, and greatly affect human health and cause many diseases due to their persistent nature in biological systems. This class of pollutants is introduced to the aquatic environment from the discharge of many industries, such as textiles, pharmaceuticals, packaging, tanning, etc. Currently, increasingly severe limits on the extent of organic pollutants have been established. There are different methods used to treat industrial waste water containing organic dyes: photo-degradation [1], reverse osmosis [2], biological treatment [3], and oxidative degradation [4]. However, most of these methods generally suffer from disadvantages such as low efficiency, extraordinary cost, the emergence of ancillary contaminants, revival problems, and long retention periods. One of the most promising and efficient methods of wastewater treatment is adsorption, which is characterized with simple utilization, low cost, and the facility to revive both adsorbent and pollutants [5,6]. It is well known that activated carbon is the most frequently used solid adsorbent for the removal of different organic and inorganic pollutants from different aquatic environments [7,8,9,10]. Nowadays, research scientists world-wide are looking for innovative classes of adsorbents characterized by high adsorption efficiency, as well as strong affinity towards certain environmental pollutants such as organic dyes. Synthetic nanoclay minerals are a new class of nanomaterial characterized by unique features, such as their intercalated anionic layered structure, which could be substituted by another cation in the interlayer gallery; purity of their structure; and their well-designed composition, which enable them to be used in different applications, such as catalysis [11,12], nanocomposite reinforcement [13,14,15], and antibacterial agents [16]. Moreover, different clays have been used for environmental remediation for the removal of heavy metals [17,18], the antimicrobial peptides nisin and pediocin [19], herbicides [20], vitamin B1 [21], and different organic dyes [22,23,24,25,26].

However, the application of synthetic nano-clay magadiite (SNCM) for the elimination and remediation of different contaminants from the water environment is rare in the literature [27,28,29]; extra effort is desired to discover the adsorption capabilities of synthetic nano-clay magadiite as an effective adsorbent for the elimination of organic dye as a specimen of organic contaminants—a main class of ecological contaminants that are persistent in the environment and cause antagonistic possessions on both fauna and flora.

Na-magadiite (Na_2_Si_14_O_29_·*n*H_2_O) is one of the most significant aluminum-free layered silicates, with an unknown exact crystal structure. However, three probable structures have been anticipated [30]. Furthermore, the layered appeal and the swelling behavior of Na-magadiite are well designated in the literature [31,32,33,34]. It is well recognized that the layers of Na-magadiite can be prolonged by suitable treatment by intercalation of guest molecules among the sheets of Na-magadiite, which opens the gate for possible adsorption affinity.

In the present work, synthetic nanoclay Na-magadiite (SNCM) was synthesized and characterized with dissimilar methods: by scanning electron microscope (SEM), surface area analyzer (SAA), and X-ray diffraction (XRD). It was then used for the elimination of methylene blue dye (MB)—as an example organic contaminant—from a model solution and a wastewater sample. The influence of the different operational parameters affecting the removal process was studied and optimized. The adsorption of the MB dye from the model solution by SNCM was revealed kinetically using altered kinetic models and thermodynamics to gain a sympathetic understanding of the adsorption process.

## 2. Experimental

### 2.1. Materials

All chemicals were of analytical grade and were obtained from Sigma-Aldrich (Aldrich, Dorset, UK). The experiments were performed using de-ionized water with a resistivity that did not exceed 18.2 MΩ·cm and which was obtained with a Millipore Milli-Q system (Billerica, MA, USA). A stock solution (100 mg∙L^−1^) was prepared by dissolving a known quantity of MB dye in de-ionized water. The stock solution was further diluted to the desired concentration for other experiments.

### 2.2. Synthesis of SNCM

SNCM was synthesized using hydrothermal treatment under autogenous pressure at 150 °C of a viscous weak alkaline and SiO_2_-rich area of the ternary system Na_2_O/SiO_2_/H_2_O [35]. The molar gel composition was 5 SiO_2_:Na_2_O:75 H_2_O. From the chemical and thermogravimetric analysis (TGA), it was found that the chemical composition of the synthesized Na-magadiite was 13.8 SiO_2_:Na_2_O:9.8 H_2_O.

### 2.3. Characterization

The XRD diffraction pattern was obtained using an X’pert Pro diffractometer from Phillips Analytical (Almelo, The Netherlands). CuK α radiation (λ = 1.54056 Å) was used. The sample was measured in the sample holder with a small exposure area. The diffraction pattern of the sample was measured by using the following program: 2θ angle from 2° to 50° with a step of 0.02° and a duration of 3 s per step. Morphology of the samples was determined by using Carl Zeiss Gemini ULTRA 55 SEM equipment at a 3 kV voltage and with an SE 2 detector (MST AG, Germany). Nitrogen-sorption measurements were taken by first carrying out a pre-treatment of the samples. During pre-treatment, the samples were heated with a 1 °C per minute rate to 250 °C under high vacuum, and kept at these conditions for 12 h. Analysis measurement was performed at a temperature of −176 °C in liquid nitrogen with Quadrasorb SI equipment (Quantachrome, Boynton Beach, FL, USA).

### 2.4. Adsorption Studies

The adsorption experiments were carried out in batch mode by mixing a specific amount of SNCM and 10 mL of MB dye solution in a stoppered conical flask under constant shaking (120 rpm) in a thermostat shaker. The effects of contact time, SNCM dosage, pH, ionic strength, and temperature of the MB dye solutions were investigated. For determination of the equilibrium time of adsorption, the experiments were carried out in specific time intervals using 0.25 mg of the SNCM and 5 mg/L MB dye solution. For the effect of SNCM dosage experiments, 1–70 mg of SNCM and 10 mL of 5 mg/L MB dye solution were used at 20 °C and a contact time of 60 min. For the effect of solution pH, 10 mg of SNCM and 10 mL of 5 mg/L MB dye solution were used at 20 °C and a contact time of 60 min. For the effect of ionic strength, 10 mg of SNCM and 10 mL of 5 mg/L MB dye solution were used at 20 °C and a contact time of 60 min. The temperature effect was investigated kinetically at three different temperatures: 20, 35, and 50 °C. After the completion of the adsorption experiment, the solution was immediately filtered to collect the supernatant, and the residual MB dye concentration in the supernatant was measured using a UV-VIS instrument at 664 nm. The removal efficiency was expressed as the percentage of MB dye removed, and the uptakes of SNCM (*q_e_*, mg/g) were calculated by Equations (1) and (2), respectively:

(1) $$\%\;\mathrm{MB}\;\mathrm{dye}\;\mathrm{removed}=\frac{\left(C_{0}-C_{e}\right)}{C_{0}}\times 100$$

(2) $$q_{e}=\frac{\left(C_{0}-C_{e}\right)\times V}{m}$$

where *C*_0_ and *C_e_* (mg/L) are the initial and equilibrium concentrations of the MB dye in solution, respectively; *V* (L) is the volume of the MB dye solution; and m (g) is the SNCM dosage.

## 3. Results and Discussion

### 3.1. Characterization of the SNCM

**Figure 1:**
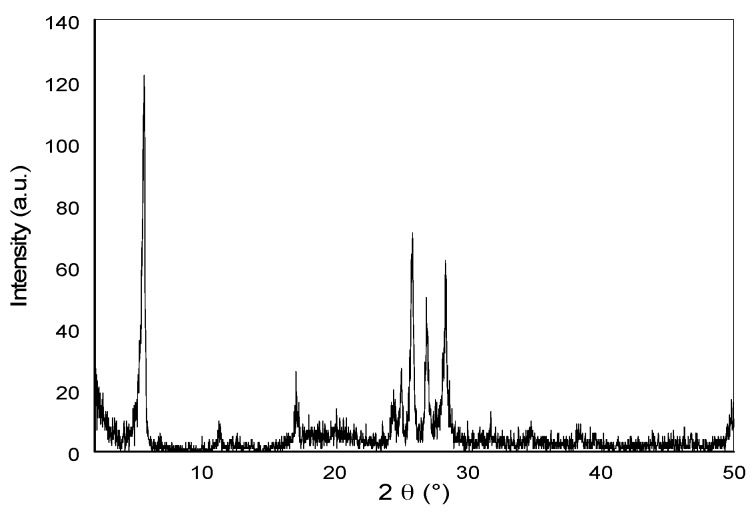
X-ray diffraction pattern of the as-synthesized layered sodium silicate magadiite.

The X-ray diffraction pattern of the as-synthesized dried sample showed the characteristic peaks of magadiite at 5.6°, 11.3°, and 17.11° 2θ corresponding to the (001), (002), and (003) diffraction planes, respectively [36]. The crystalline nature of the magadiite phase layer was indicated by the peaks found between 23° and 30° 2θ. In addition, a basal spacing of 1.54 nm corresponding to the *d*_001_ reflection was detected, as shown in Figure 1 [37]. The average crystallite size was 16.63 nm, and the lattice strain was 0.0416 as deduced from Scherrer equation [38]. The small crystallite size of SNCM indicates the presence of the anionic sheets as nano-sheets.

**Figure 2:**
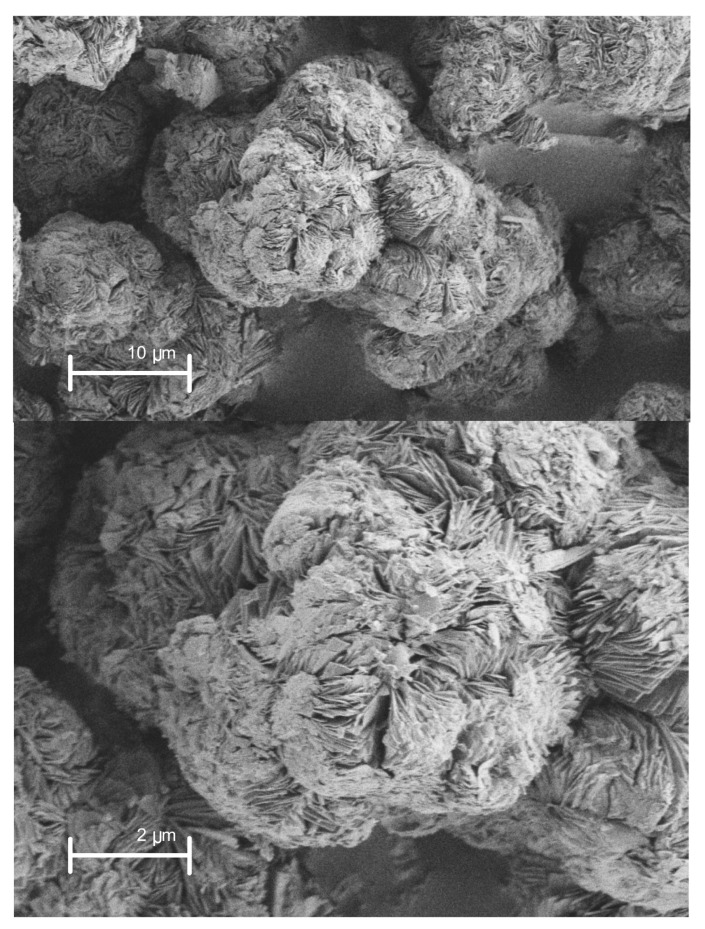
SEM image of as-synthesized layered sodium silicate magadiite.

SEM image (Figure 2) of SNCM showed well-crystallized rosette-shaped Na–magadiite particles (SEM), with a diameter for the “flower” and “petals” of about 5–7 µm. The single magadiite crystalline phase was obtained as a result of the controlled hydrothermal reaction temperature at 150 °C. It was reported in the literature that the effect of the temperature is much more important than the reaction time and concentration in order to synthesize single magadiite phase, and the reaction temperature must be below 170 °C [39].

**Figure 3:**
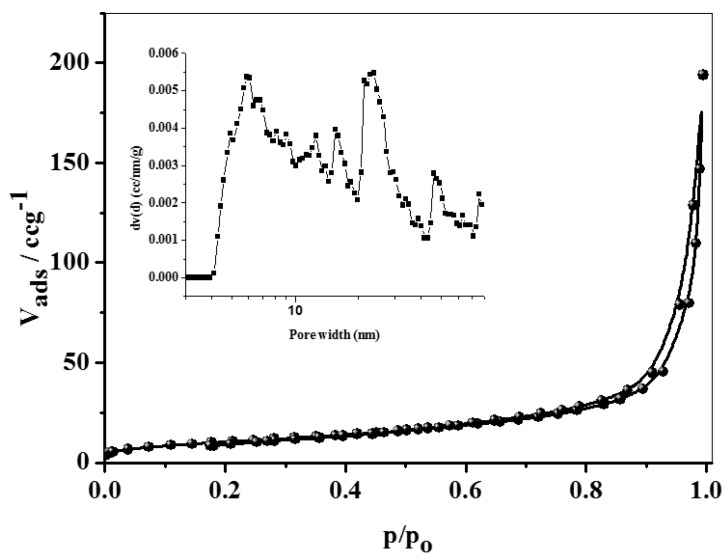
The N_2_ sorption isotherm and pore size distribution curve for SNCM.

The N_2_ sorption isotherm and Barrett-Joyner-Halenda (BJH) pore size distribution shown in Figure 3 were obtained for SNCM sample. The preliminary monolayer–multilayer adsorption on the mesopore walls receives a similar track to the type II isotherm, and is shadowed by pore condensation with H_3_ hysteresis according to IUPAC classification in 1985 [40]. This loop shape is due to the non-rigid aggregates of Na-magadiite plate-like particles and macropores which are not entirely occupied with pore condensate [41]. SNCM has a BET surface area of 39 m^2^·g^−1^ and a total pore volume of 0.3 cm^3^·g^−1^. The small surface area of Na-magadiite is identical to the alkaline magadiite reported elsewhere [42]. Surface areas of magadiite could not be entirely attributed to the exterior surface due to the layered structure, in which the nitrogen has contact. Although the low BET surface area of SNCM could negatively affect the adsorption capacity towards the organic dye, the intercalation ability between the anionic sheets of magadiite could compensate for this plausible effect. The multi-modal pore size structure is shown in the inset of Figure 3. The average pore size of SNCM is ca. 28 Å.

### 3.2. Effect of Operational Parameters

**Figure 4:**
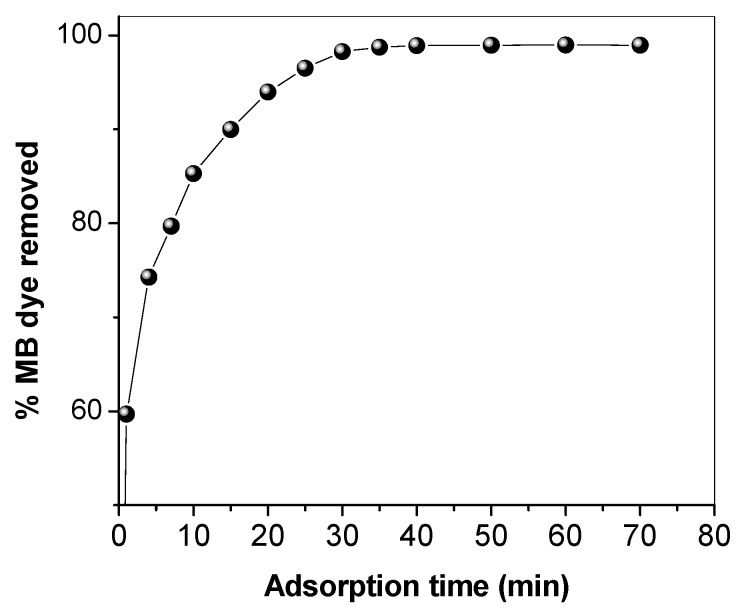
Effect of adsorption time on the percentage of methylene blue dye (MB) removed from an aqueous solution by synthetic nano-clay magadiite (SNCM).

**Figure 5:**
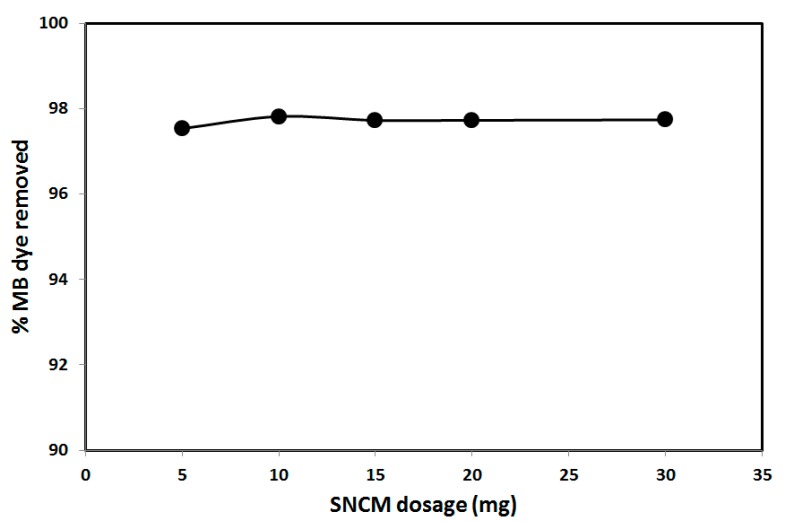
Effect of the SNCM dosage on the removal of methylene blue dye from an aqueous solution.

Environmental and operational parameters greatly affect the removal and adsorption of pollutants from water using any solid adsorbent. Therefore, the study of adsorption contact time, SNCM dosage, solution pH, and ionic strength are of great importance. The effect of time on the adsorption of MB dye from an aqueous solution by SNCM was studied to explore the time required to reach equilibrium, and the results are presented in Figure 4. As shown in Figure 4, the percentage of MB dye removed from the solution reached 46.3% within 1 min and significantly increased to 90.3% after 25 min, and to 91.0% within 30 min A further increase in the contact time did not significantly alter the percentage of MB dye removed, which reached 94.0% after 60 min, and consequently, a contact time of 30 min was selected for further studies. Figure 5 shows the effect of the SNCM dosage on the removal of MB dye from aqueous solution. It is clear that most of the MB dye was removed from the solution using 5 mg of the SNCM; 97.5% of the MB dye was removed, and this removal percentage did not change with a further increase in the SNCM dosage. This means that 5 mg of the SNCM was adequate to remove the MB dye completely from the solution, and this level was selected for further studies.

**Figure 6:**
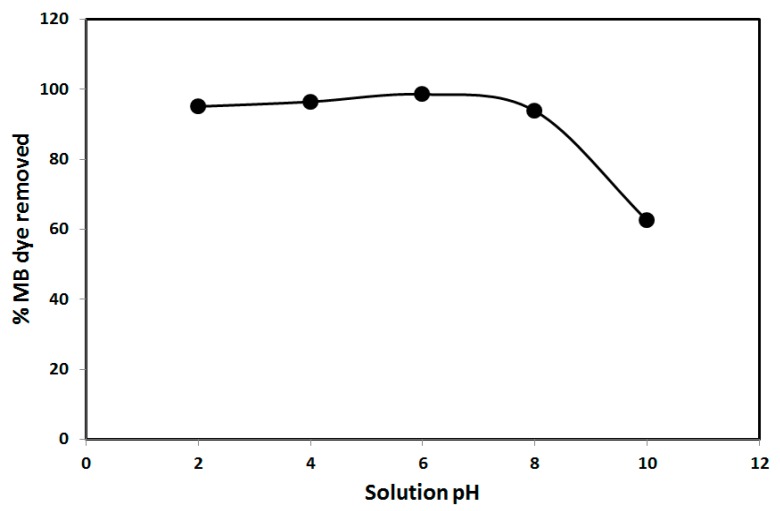
Effect of solution pH on the percentage of methylene blue dye removed from an aqueous solution by SNCM.

The effect of solution pH on the removal of MB dye by SNCM was investigated due to its significant effect on the adsorption process, and the results are presented in Figure 6. The results show that increasing the solution pH was accompanied by a slight enhancement in the percentage of MB dye removal: 95.1%, 96.4%, and 98.6% at pH values of 2, 4, and 6, respectively. This indicated the insignificant competition between the hydronium ions (H_3_O^+^) and the MB molecules for the adsorption on the SNCM surface at low pH values. A further increase in the solution pH to 8 was associated with a slight decrease in the percentage of MB dye removed, where it decreased to 93.8%, which significantly decreased to 62.4% upon increasing the solution pH to 10. This decrease in the adsorption capacity upon elevating the pH values could be attributed to competition between the MB dye molecules and the hydroxyl ions present at these pH values, as the MB dye molecules are insensitive to the pH change [43]. Additionally, the point of zero charge for the SNCM was measured and was 5.1, indicating that the SNCM surface is negatively charged at pH values higher than 5.1, which accordingly creates electrostatic repulsion with the negatively-charged MB (with a *p*K_a_ value of 3.14), which agrees well with previous results [29,44]. The effect of ionic strength on the removal of organic pollutants such as MB dye from water by solid adsorbents such as SNCM is significant, because it sheds light on the nature of the interactions between the MB dye molecule and the SNCM surface, and explains if it is electrostatically attractive or repulsive [28].

**Figure 7:**
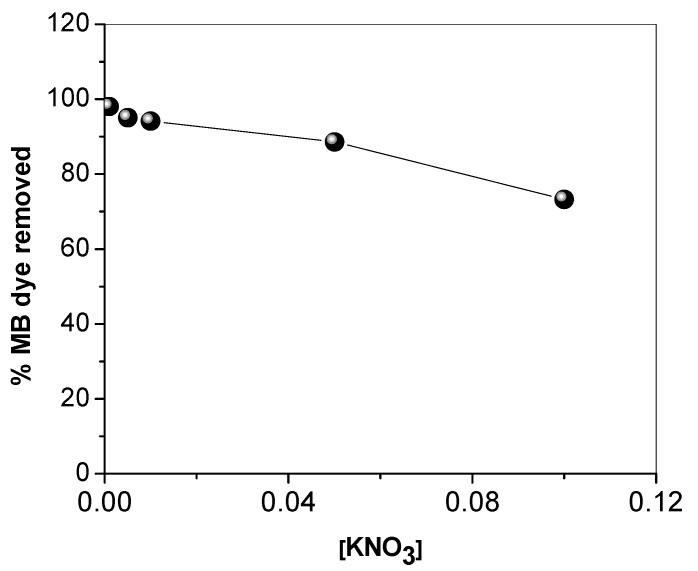
Effect of solution ionic strength on the percentage of methylene blue dye removed from an aqueous solution by SNCM.

The effect of the ionic strength on the removal of MB dye from water using SNCM was studied using different concentrations of KNO_3_, and the results are presented in Figure 7. It is clear that the percentage of MB dye removed was insignificantly affected by an increase in KNO_3_ from 0.005 to 0.01 M: 95.0% and 94.2%, respectively. A further increase in KNO_3_ concentration to 0.05 and 0.1 M caused the decrease in the percentage of MB dye removed to 88.6% and 73.2%, respectively. This may be due to the hindering effect of the high concentration of K^+^ and NO_3_^−^ ions, indicating the electrostatic nature of the MB dye adsorption by the SNCM surface.

### 3.3. Kinetics and Thermodynamics Studies

**Figure 8:**
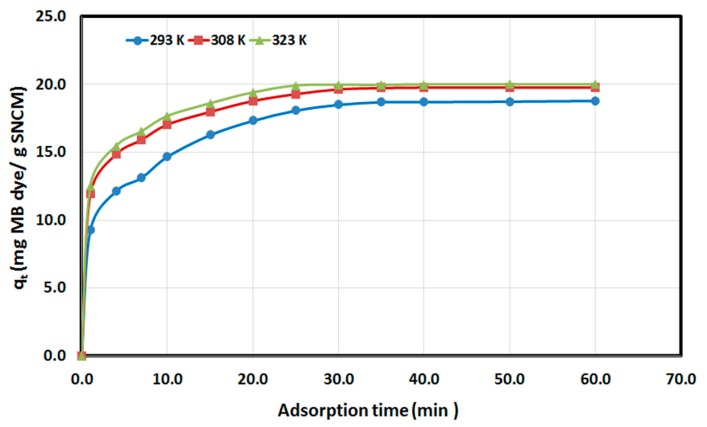
Variation of the amount MB dye adsorbed per unit mass of SNCM (*q_t_*) with time.

The variation of the amount of MB dye adsorbed by SNCM as a function of the adsorption time was studied at three different temperatures—293, 308 and 323 K—and the experimental results are shown in Figure 8. It is clear from the figure that the amount of MB dye removed from solution by SNCM reached equilibrium within 30 min: 18.5, 19.6, and 19.9 mg MB/g SNCM at 20, 35, and 50 °C, respectively, and no significant improvement in the amount of MB dye removed was observed with a further increase in time. Additionally, it was clear that the adsorption was endothermic in nature, as the amount of MB dye removed was enhanced with increasing solution temperature: 18.8, 19.8, and 20.0 mg MB/g SNCM at 20, 35 and 50 °C after 60 min, respectively.

**Figure 9:**
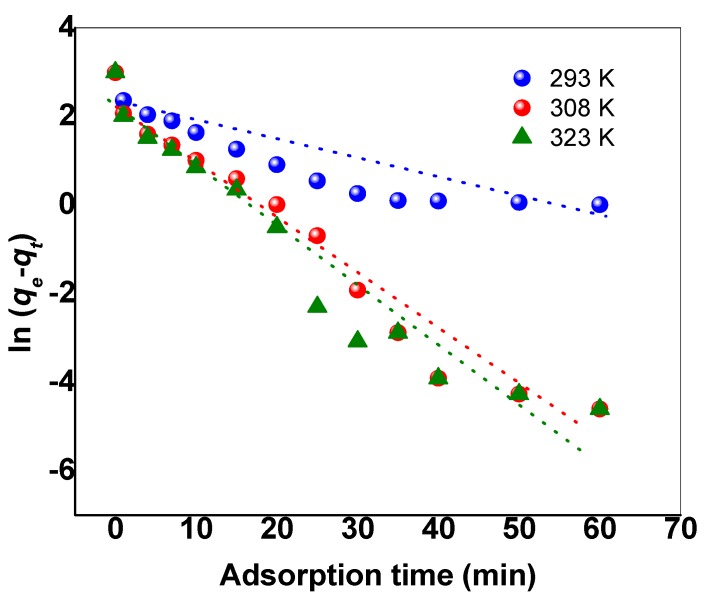
The application of the pseudo-first-order kinetic model for the MB dye removed from an aqueous solution by SNCM.

**Figure 10:**
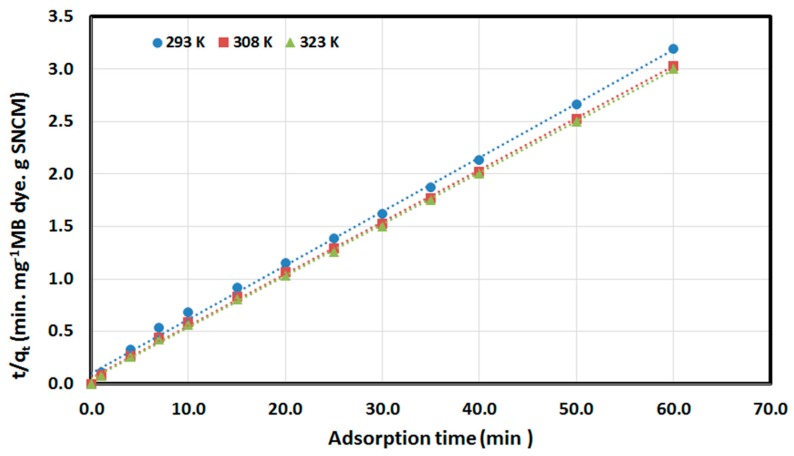
The application of the pseudo-second-order kinetic model for the MB dye removed from an aqueous solution by SNCM.

The experimental results of the effect of contact time on the removal of MB dye by SNCM were used to study the adsorption kinetics using the most used kinetic models—namely, the pseudo-first-order kinetic model and the pseudo-second-order kinetic model, as presented in Equations (3) and (4), respectively:

(3) $$\ln(q_{e}-q_{t})=\ln q_{e}-k_{1}t$$

(4) $$\frac{t}{q_{t}}=\frac{1}{k_{2}q_{e}^{2}}+\frac{t}{q_{e}}$$

where *q_e_* and *q_t_* are the values of the amount MB dye adsorbed per unit mass of SNCM at equilibrium and at any time *t*, respectively; *k*_1_ (min^−1^), *k*_2_ (g/(mg∙min)) are the pseudo-first-order adsorption rate coefficient, and pseudo-second-order rate coefficient, respectively. Applying the pseudo-first-order kinetic model (Equation (3)) to the experimental results (Figure 9), the plot of ln(*q_e_* − *q_t_*) vs. *t* for MB dye at different temperatures did not converge well and did not give straight lines (as is clear from Figure 9), with unacceptable regression coefficients. Additionally, the estimated values of the amount adsorbed at equilibrium (***q****_e_***_,_***_calc_*) were far from the experimental values (***q_e_*_,_***_exp_*). This indicated that pseudo-first-order kinetic model is not appropriate for the description of MB dye removal by SNCM from water. Applying the pseudo-second-order kinetic model (Equation 4) to the experimental data, the plot of *t/q_t_* vs. *t* converged well, with straight lines and an excellent regression coefficient higher than 0.99, as presented in Figure 10. In addition, there was an excellent correlation between the calculated amount of MB adsorbed by the SNCM (***q_e_*_,*calc*_**) and the experimental values (***q_e_*_,*exp*_**). These findings confirmed the suitability of the pseudo-second-order kinetic model for describing the removal of MB dye from the model solution by SNCM.

There are many previous studies that also showed the suitability of the pseudo-second-order kinetic model for the description of MB dye from water by the different solid adsorbents, such as Fe_3_O_4_-graphene@mesoporous SiO_2_ nanocomposites [45], zeolite synthesized from electrolytic manganese residue [46], zinc oxide nanorods loaded on activated carbon [47], activated carbon [48], poly(sodium p-styrene sulfonate)/poly(methyl methacrylate) particles [49], and many other adsorbents. The thermodynamic parameters include the enthalpy change (Δ*H*), free energy change (Δ*G*), and entropy change (Δ*S*), and were calculated to evaluate the thermodynamic feasibility and the spontaneous nature of the MB dye removal by SNCM according to the following equations:

(5) $$D=\frac{q_{e}}{C_{e}}$$

(6) $$\ln\;D=\left(\frac{\Delta S}{R}\right)-\frac{\Delta H}{R}\times \frac{1}{T}$$

**Figure 11:**
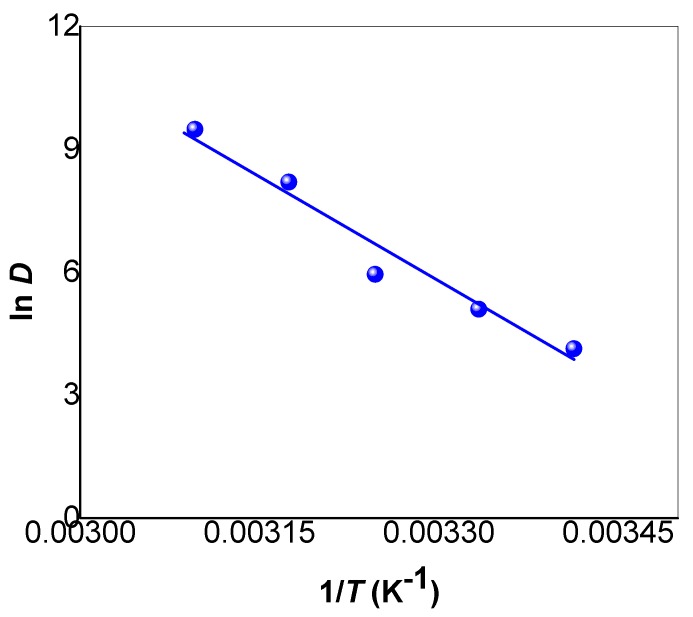
Plot of ln *D* vs. 1/*T* for the MB dye removed from an aqueous solution by SNCM.

(7) ΔG=ΔH−T ΔS

where *D* is the distribution coefficient; *R* is the gas constant (8.314 J·mol^−1^·K^−1^); and *T* is the temperature (K). The values of Δ*H* and Δ*S* are determined from the slope and the intercept of the plots of ln *D* versus 1/*T*, which is associated with a good correlation coefficient; R^2^ equal 0.961, as is shown in Figure 11. The removal of MB dye using SNCM from water associated with Δ*H* value of +144.0 kJ/mol, indicating the adsorption process was endothermic in nature. This finding confirmed the above-mentioned result that the adsorption is fast and obeys the pseudo-second-order kinetic model. The Δ*S* value of +523 J/mol∙K indicates the increase in the degree of disorder upon the adsorption of the MB dye molecules by the SNCM. The Δ*G* value was calculated based on Equation (7) at 20 °C, and the value was found to be negative (–9.18 kJ/mol), indicating that the process was spontaneous, and this value became more negative by raising the solution temperature: –16.9 kJ/mol and −24.6 kJ/mol at 35 °C and 50 °C, respectively. It could be concluded here that the more negative the Δ*G* value, the more spontaneous the removal, which was accompanied by higher values of MB dye uptake by the SNCM. Additionally, the negative values of Δ*G*, the positive value of Δ*H*, and the positive value of Δ*S* indicated that the removal of MB dye by SNCM is an entropy-driven process. According to the kinetics and thermodynamics study, the MB dye removal by SNCM could be described by the pseudo-second-order kinetic model, and was spontaneously endothermic and chemical in nature.

According to the above results, the adsorption capacity of MB by SNCM at ambient temperature is 18.8 g MB/g within 30 min. In comparison with other adsorbents, SNCM could be considered as a potential and promising adsorbent for the removal of organic dyes, such as MB from water. This adsorption capacity is much higher and better compared with spent rice biomass: 8.13 mg MB/g [50]; activated carbon prepared from rice husk: 9.73 mg MB/g [51]; natural Jordanian Tripoli: 16.6 mg MB/g [52]; and lower compared with manganese oxide nanocorals: 41.26 mg MB/g [46]; and surface hydroxyl group-enriched TiO_2_ nanotubes: 57.14 mg/g [53]. In general, based on the above results, it could be stated that SNCM is a promising and potentially competitive adsorbent for the removal of organic dyes such as MB from solution.

## 4. Recycle Study

The recycle and reuse of the SNCM was studied. The desorption process was performed by soaking and washing the SNCM sample in acetone, then drying and reusing it for the removal of MB from solution. The percentage of MB removed was 98.1% using 5 mg of SNCM, and this value did not change significantly after four cycles of reuse. This showed the great ability to recycle and reuse SNCM many times without losing adsorption efficiency.

## 5. Conclusions

The removal of the organic dye methylene MB from water was studied using SNCM. Firstly, SNCM was synthesized by hydrothermal treatment under autogenous pressure and then characterized using different techniques in order to explore its physical and morphological structure. The XRD results showed the characteristic peaks of magadiite with average crystallite size 16.63 nm, and the SEM images showed well-crystallized rosette-shaped Na–magadiite particles with diameters for “flower” and “petal” of about 5–7 µm, and BET-surface area 33 m^2^·g^−1^. The effects of different operational and environmental parameters, such as removal time, SNCM dosage, contact time, pH, and ionic strength of the solution, on the removal of MB dye were explored. The adsorption results showed the great affinity of the SNCM to the MB dye molecules, which reached an adsorption uptake of 20.0 mg MB dye/g of SNCM. The removal of MB dye by SNCM was studied kinetically and thermodynamically, was found to follow the pseudo-second-order kinetic model, and was spontaneous and endothermic in nature. Finally, SNCM showed that it can be considered as a promising adsorbent for the removal of MB dye from an aqueous solution.
